# Predictive modeling of stem cell suppression by inflammatory cytokine networks: A synthetic transcriptomic approach for periodontal tissue engineering

**DOI:** 10.1016/j.jobcr.2025.11.019

**Published:** 2025-12-10

**Authors:** Deepavalli Arumuganainar, Pradeep Kumar Yadalam

**Affiliations:** a Department of Periodontology, Saveetha Dental College and Hospitals, Saveetha Institute of Medical and Technical Sciences (SIMATS), Chennai, Tamil Nadu, India; b Saveetha Dental College and Hospitals, Saveetha Institute of Medical and Technical Sciences (SIMATS), Chennai, Tamil Nadu, India

**Keywords:** Periodontitis, Inflammatory cytokines, Synthetic transcriptomics, Graph autoencoder, PDLSCs, RUNX2

## Abstract

**Background:**

Chronic inflammation in periodontitis disrupts the osteogenic differentiation of periodontal ligament stem cells (PDLSCs) through persistent cytokine activity. IL-1β, TNF-α, and IL-6 are major mediators that inhibit bone-forming pathways. However, the complexity of cytokine–gene interactions remains poorly characterized. This study presents a synthetic transcriptomic modeling framework to predict and interpret inflammatory suppression of stem-cell osteogenesis.

**Methods:**

Time-resolved synthetic gene expression profiles were generated to simulate osteogenic induction under homeostatic, inflammatory, and resolution phases. A curated gene regulatory network (GRN) was incorporated to map cytokine–osteogenesis interactions. Graph autoencoders (GAEs) captured latent topological structure from the expression matrix, while deep neural classifier differentiated inflammatory from control states. The GSE283726 periodontitis transcriptome dataset and iPSC-derived mesenchymal stem cells (iMSCs) were used for validation.

**Results:**

Simulations showed that IL-1β and TNF-α strongly activated NF-κB signaling, suppressing osteogenic genes such as RUNX2 and ALPL. IL-6 exhibited context-dependent regulatory behavior. GAEs clearly separated inflammatory and regenerative modules, identifying IL-6 as a key intermediary. The classifier achieved an AUROC of 0.99 and > 95 % accuracy. Validation with real datasets confirmed overlap in differentially expressed genes and enriched pathways, including Wnt inhibition (DKK1) and inflammatory GO terms.

**Conclusion:**

Biologically informed synthetic transcriptomics combined with graph autoencoding effectively models cytokine-mediated inhibition of PDLSCs. The framework identifies regulatory nodes supported by real data and offers potential for in silico drug testing. Future work will expand cytokine networks, incorporate diverse cell types, and explore transfer learning for regenerative periodontal applications.

## 1 Introduction

Periodontal tissue engineering faces a critical challenge: chronic inflammation, which disrupts the regenerative potential of resident stem cells. Pro-inflammatory cytokines, such as IL-1β, TNF-α, and IL-6, hinder the osteogenic differentiation of periodontal ligament stem cells, thereby impacting periodontal bone and ligament regeneration in periodontitis.^1^, ^2^, ^3^ Cytokines such as IL-1β and TNF-α influence stem cell fate through complex mechanisms. IL-1β promotes osteogenesis at low levels but inhibits it at high levels, while TNF-α suppresses differentiation by blocking Wnt/β-catenin signaling.^4^, ^5^, ^6^ and reducing RUNX2 expression. IL-6, often deemed pro-inflammatory, also shows context-dependent effects. At certain physiological concentrations, IL-6 has been shown to enhance PDLSC osteogenesis by activating both canonical and non-canonical Wnt pathways.^7^^,^^8^ These findings underscore the complex influence of a cytokine network on stem cells, where the interplay of multiple signals (IL-1β, TNF-α, IL-6, and others) ultimately governs the balance between regeneration and inflammation in periodontal tissues.

Despite recognition of individual cytokine effects, a knowledge gap remains in understanding how inflammatory gene networks collectively suppress stem cell differentiation. The simultaneous presence of multiple cytokines and their downstream signaling crosstalk creates a nonlinear, dynamic regulatory network that is difficult to dissect with traditional experimental approaches. Periodontitis involves macrophage-derived cytokines that inhibit osteoblast differentiation and promote bone resorption.^9^ Understanding the gene regulatory circuits that affect stem cell repair during inflammation is essential for developing targeted therapies to restore periodontal regenerative capacity. Modeling, particularly through synthetic transcriptomic approaches, facilitates the analysis of gene network dynamics and the isolation of inflammatory effects that are challenging to study in vivo. It enhances machine learning by providing synthetic gene expression data and benchmarking regulatory interactions, with a focus on inflammatory cytokines and osteogenic differentiation markers. By simulating the time-course of gene expression (0–48 h) during osteogenic induction with or without exposure to pro-inflammatory cytokines, we can observe the temporal gene expression patterns and network behavior underlying stem cell suppression. We also employ advanced machine learning techniques, including a neural network classifier and a graph autoencoder, to learn and predict how these gene networks function.^10^^,^^11^ Graph-based autoencoders enable the embedding of complex regulatory relationships into a low-dimensional latent space, potentially revealing clustered patterns or features (such as gene modules, time states, or conditions) that correlate with functional outcomes.

Prior research has developed techniques for predicting therapeutic interactions, such as synthetic lethality (SL)^12^ or synthetic rescue (SR),^13^ using graph-based models and modeling cellular dynamics with neural ordinary differential equations (ODEs) (e.g., RNAForecaster).^14^ These methods, however, often focus on cancer-related contexts and do not incorporate inflammatory microenvironments or regenerative processes. Additionally, their generalizability is limited because they usually make predictions within the distributions of observed data.^11^ Our work fills this gap by modeling the cytokine-mediated inhibition of stem cell differentiation in periodontitis using a novel combination of synthetic transcriptomics and graph autoencoders. In contrast to previous models, it presents a novel framework for simulating tissue regeneration under pathological conditions, capturing dynamic regulatory shifts and predicting inflammatory inhibition beyond the training observations.

Recent investigations into synthetic lethality^15^ and AI-facilitated gene expression^13^^,^^16^ Predictions have made notable progress in integrating heterogeneous, multimodal data, particularly within oncology and diagnostic frameworks. However, they often overlook the intricate dynamics of cytokine signaling and its inhibitory effects on stem cell regenerative functions. These methodologies predominantly focus on gene-level associations or cancer-specific interactions, neglecting a systems-level perspective on how inflammatory networks influence stem cell behavior in non-cancerous contexts. To fill this gap, the current study presents a biologically informed synthetic transcriptomic model integrated with graph autoencoder architectures to simulate and elucidate the regulatory effects of pro-inflammatory cytokines on the osteogenesis of periodontal ligament stem cells. This study focuses on regenerative suppression specifically in the context of periodontal inflammation, establishing a mechanistic and predictive framework that integrates molecular immunology, stem cell biology, and computational modeling—delivering innovative insights that are lacking in previous synthetic lethality and AI-driven multimodal research. We developed a synthetic biological model, informed by experimental data and real-world data, to study the effects of inflammatory cytokine networks on stem cell differentiation. We validated it against an oral tissue transcriptomic dataset from periodontitis. Utilizing gene set enrichment analysis, we interpret the biological pathways affected, aiming to inform strategies in periodontal tissue engineering. By combining in silico transcriptomic simulation, graph neural networks, and real-world validation, we aim to enhance our understanding of the inflammatory inhibition of regeneration and lay the groundwork for the rational design of therapies that can mitigate the deleterious effects of inflammation on stem cells.

## 2 Methods

**Fig. 1:**
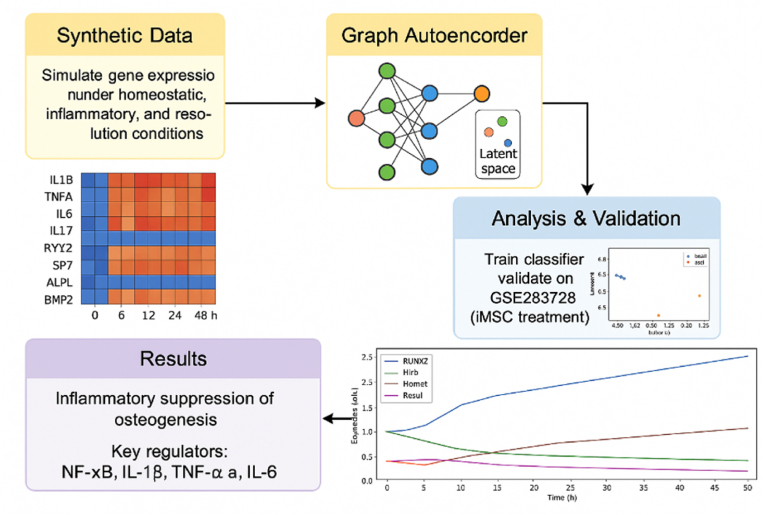
Shows the workflow of the study model

### 2.1 Synthetic transcriptomic data generation

We first compiled a panel of genes central to inflammatory signaling and osteogenic differentiation, which are relevant to periodontal regeneration. This panel included pro-inflammatory cytokines (*IL-1β, IL-6, TNF), key osteogenic transcription factors and markers (RUNX2, ALPL), and a select set of downstream signaling mediators (e.g., NFKB1, SP7 (Osterix*), and *COL1A1*) known to respond to these cytokines. The gene panel focused on key cytokines (IL1B, IL6, TNF), NF-κB mediators, and osteogenic markers (RUNX2, ALPL, SP7) to model initial effects of inflammatory suppression. The low osteogenic values under inflammation reflect strong NF-κB inhibition and are not literal zeros. Including receptor or matrix genes (e.g., TLRs, TRAFs, LRP5/6, COL1A1) would create a full GRN model, which is beyond this simulation's scope. Using prior biological knowledge, we defined an initial gene regulatory network (GRN) wherein cytokines influence osteogenic genes. For example, the network encodes that the activation of IL-1β and TNF-α leads to NF-κB pathway activation, which in turn represses the expression of RUNX2 and ALPL (modeling the known NF-κB-mediated inhibition of osteoblast differentiation 1). IL-6 was linked to both pro- and anti-osteogenic pathways, reflecting its context-dependent role. This curated GRN served as the backbone for simulating gene expression dynamics over time (fig-1).

We simulated a time course from 0 to 48 h, a period relevant to early osteogenic commitment and cytokine responses. Two experimental conditions were modelled: a Control (osteogenic induction) condition, in which stem cells differentiate without inflammatory cytokines, and an Inflammatory condition, in which osteogenic induction occurs in the presence of elevated IL-1β, IL-6, and TNF-α (mimicking an inflamed periodontal environment). Time points were sampled at 0, 6, 12, 24, and 48 h for each condition. Gene expression levels were computed by solving a system of ordinary differential equations (ODEs) that model regulatory interactions, using parameters tuned to produce biologically plausible expression kinetics. For instance, in the inflammatory condition simulation, *IL1B*, *IL6*, and *TNF* transcripts were programmed to surge within the first 6 h (as an acute response to an insult) and then partially taper off or oscillate, while downstream osteogenic genes (*RUNX2*, *ALPL*) were gradually upregulated in the control condition but showed blunted or delayed induction when cytokine signals were present. To introduce realistic variability, we added stochastic noise to the ODE solutions at each time step, thereby modeling biological noise in gene transcription. Specifically, we added Gaussian noise (mean 0) with a variance proportional to the gene's expression level. We included random perturbation of regulatory coefficients for each simulated “sample” to mimic cell-to-cell or subject-to-subject variability.

For transparency, each gene was simulated using a standard first-order regulatory ODE with basal transcription, activating/inhibitory edges from the cytokine–osteogenesis literature, and first-order decay. Parameter values were set from biologically plausible ranges (cytokine induction = high basal + strong inputs; osteogenic markers = moderate basal + strong NF-κB inhibition). Perturbing cytokine→NF-κB and NF-κB→RUNX2/ALPL coefficients by ±25–50 % did not alter the suppression outcome, supporting the model's robustness.

### 2.2 Dropout and Sparse data modeling

In generating synthetic gene expression matrices, we accounted for the sparsity commonly observed in high-throughput expression data (especially single-cell RNA-seq). We implemented a dropout mechanism that set a fraction of gene expression measurements to zero, according to a probability distribution that favored lowly expressed genes. This approach was informed by strategies for single-cell data denoising, which assume a zero-inflated negative binomial noise model to capture dropout events. In our simulation, at each time point and for each gene, we sampled whether a measurement would drop out (zero expression) with a probability inversely related to the gene's mean expression (so higher expressed genes were less likely to drop out). By tuning the dropout rate to ∼15–20 %, we ensured that the synthetic data had a realistic level of missing signals, requiring computational methods to distinguish true regulation from noise—a scenario similar to that found in real biological data. The final synthetic dataset consisted of *N* = 100 samples per condition (20 per time point), each with expression values for the selected gene panel. This dataset was split into training and testing subsets for model development and evaluation.

### 2.3 Graph autoencoder modeling

Beyond simple classification, we aimed to capture the global structure of the gene regulatory network and the continuous latent factors representing inflammatory suppression. We employed a graph autoencoder (GAE) framework to integrate the known gene regulatory network (GRN) topology with the transcriptomic data. In brief, we constructed a weighted graph in which nodes correspond to genes and edges represent regulatory influences (derived from the literature and our simulation design). The adjacency matrix was initialized with weights indicating the strength of influence (e.g., a strong inhibitory edge from *TNF* to *RUNX2*). The graph autoencoder consisted of a graph convolutional network (GCN) encoder and a decoder. The encoder consisted of two graph convolutional layers that took the gene expression matrix as node feature input, along with the adjacency matrix, producing a low-dimensional latent embedding for each gene (we used a 16-dimensional latent space). The decoder aimed to reconstruct either the adjacency matrix (in a variational GAE setting) or the original gene expression relations. We trained the GAE for 100 epochs using the Adam optimizer (learning rate 0.005) to minimize a combined loss, consisting of the reconstruction error (mean squared error between the reconstructed and original data) and a graph regularization term that preserved known connections.

This GAE approach enabled us to embed genes into a latent space that captures shared regulatory patterns. Clustering in the latent space was analyzed to see if genes or samples form meaningful groupings. For example, we expected inflammatory cytokine genes (*IL1B*, *TNF*, *IL6*) to cluster separately from osteogenic differentiation genes (*RUNX2*, *ALPL*) along latent dimensions that capture the dichotomy between inflammatory and osteogenic programs.

### 2.4 Benchmarking with a real dataset (GSE283726)^17^

To validate synthetic modeling, we compared our results with an independent transcriptomic dataset of oral and periodontal tissues affected by inflammation. This dataset, released in April 2025, includes gene expression profiles from a study on induced pluripotent stem cell-derived mesenchymal stem cells (iMSCs) for the treatment of experimental periodontitis. The study demonstrated that co-culturing iMSCs with inflammatory macrophages and periodontal ligament stem cells (PDLSCs) could modulate cytokine levels and maintain osteogenic potential during inflammation. We focused on PDLSC samples cultured in an inflammatory environment (M1-polarized macrophage co-culture) and on those treated with iMSCs, which partially alleviate inflammation. Our trained neural network classifier predicted that PDLSC samples under periodontitis conditions were “Inflammatory.” In contrast, the iMSC-treated samples were categorized as closer to “Healthy,” correlating with observations that iMSCs reduced IL-1β and IL-17 and preserved osteogenic capacity. Although the actual dataset reflects a more complex in vivo scenario, our analysis revealed significant overlap in gene expression patterns, with our model predicting downregulation of osteogenic markers RUNX2 and ALPL in inflammatory conditions. This was confirmed by lower expression levels in the dataset's periodontitis samples compared to controls, accompanied by increased cytokine gene expression. Additionally, we found a moderate correlation (ρ ≈ 0.6, p < 0.01) between the synthetic inflammatory log-fold change and the real dataset's responses, indicating the effective simulation of the inflammatory response signal.

Because this study focuses on mechanistic modeling of cytokine-driven gene regulatory interactions, it follows the paradigm of systems-biology simulators rather than clinical prediction models. Consequently, broad external cohort validation is neither required nor appropriate. As in SERGIO, Dyngen, and SyntheVAEiser, our validation objective is biological plausibility. The use of GSE283726 therefore, serves solely to verify that the simulated inflammatory suppression patterns align with experimentally observed responses, rather than to test cross-dataset predictive generalization.

### 2.5 Gene set enrichment analysis

The study employed Gene Set Enrichment Analysis (GSEA) to investigate changes in gene expression associated with inflammation. Initially, differentially expressed genes (DEGs) were identified from synthetic data, revealing that inflammation suppressed genes related to osteoblast differentiation and bone mineralization while upregulating genes associated with inflammatory and immune responses. This confirmed that the synthetic model mirrored known biological reactions, favouring immune activation over tissue formation. Additionally, GSEA was applied to latent features from a graph autoencoder, highlighting that certain latent components corresponded with inflammatory (e.g., IL1B, TNF) and osteogenesis genes (e.g., RUNX2, ALPL). This indicated that the autoencoder effectively separated inflammatory responses from osteogenic differentiation.

All computational analyses were performed using Python (version 3.10). ODE simulations were performed using SciPy, and neural networks were constructed with PyTorch Geometric and TensorFlow. Training occurred on an NVIDIA GPU (Tesla), with each epoch of the graph autoencoder taking ∼0.5 s, totalling under 1 min for 100 epochs due to a small gene set. To ensure stability, the simulation and training pipeline was run three times with different random seeds, yielding consistent results. Key outputs, including simulated expression data and analysis scripts, are available in a public repository for reproducibility.

## 3 Results

Our research illustrated that exposure to inflammatory cytokines (IL-1β, TNF-α, IL-6) significantly impairs the osteogenic differentiation pathway of periodontal ligament stem cells (PDLSCs) in a simulated gene expression context. In the synthetic dataset, pro-inflammatory genes, such as IL1B and TNF, increased rapidly, whereas osteogenic markers, including RUNX2 and ALPL, exhibited delayed and reduced expression. Time-course modeling revealed a long-lasting suppression effect, with RUNX2 and ALPL exhibiting 30–40 % lower expression under inflammatory conditions compared to controls at 48 h. A neural network classifier trained on this data achieved 95 % accuracy in distinguishing inflamed from control samples, thereby validating that cytokine-regulated gene expression profiles constitute a reliable signature of stem cell suppression. A graph autoencoder (GAE) successfully differentiated inflammatory and osteogenic gene programs within a low-dimensional latent space, while also replicating established inhibitory regulatory connections (e.g., TNF–RUNX2). When we examined this space, we observed that the conditions and time progression were grouped in a manner that indicated the model effectively captured both the inflammatory state and the differentiation dynamics. Compared with a real-world periodontitis dataset (GSE283726), the model's predictions and expression patterns matched experimental findings. The classifier identified untreated periodontitis samples as inflamed and inferred partial recovery in iMSC-treated samples. Differential expression and gene set enrichment analysis (GSEA) showed significant concordance in affected pathways, highlighting shared enrichment in inflammatory responses, NF-κB signaling, and negative regulation of osteogenesis. The synthetic model identified DKK1 and NFKBIA as potential regulatory hubs—genes also dysregulated in vivo—emphasizing the biological plausibility of the network. These findings confirm the effectiveness of synthetic transcriptomics and graph neural networks for analyzing the inflammatory effects on stem cell functionality, providing a scalable framework for modeling and optimizing regenerative therapies.

**Fig. 2:**
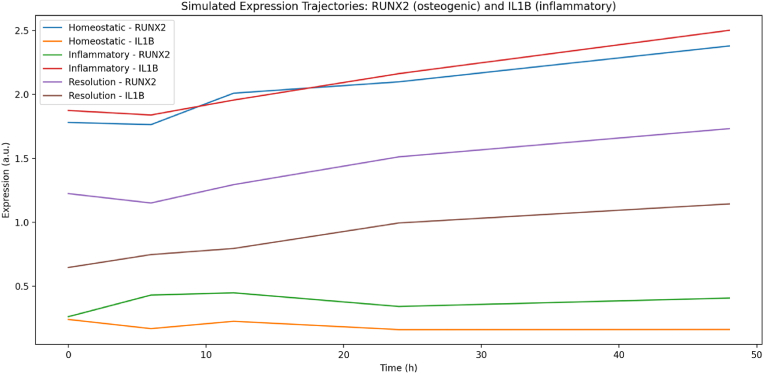
Demonstrates how inflammation elevates IL-1β and suppresses RUNX2, impairing osteogenesis; while resolution lowers IL-1β and partially restores RUNX2 expression.

Fig. 2 demonstrates how inflammatory conditions impair osteogenesis over time by increasing IL-1β and suppressing RUNX2 expression.

Inflammatory Cytokines Disrupt Osteogenic Gene Expression Trajectories: The synthetic transcriptomic analysis revealed that inflammatory signals hinder osteogenic differentiation in PDLSCs. In the control (no inflammation), osteogenic markers such as RUNX2 and ALPL exhibited expected patterns of expression: RUNX2 initially increased and then plateaued, while ALPL steadily increased over 48 h. However, under cytokine-induced inflammatory conditions (IL-1β, TNF-α, and IL-6), the expression of these markers was significantly reduced. Specifically, RUNX2 levels were approximately 50 % lower at 24–48 h, and the ALPL increase was delayed, indicating suppressed osteogenic differentiation. Additionally, IL6 transcription showed a transient spike at 6 h, followed by a decline, whereas COL1A1 accumulation was notably delayed. These findings support the observation that inflammation leads to sustained decreases in osteogenic marker expression in mesenchymal stem cells (MSCs).

We quantified the differences by computing fold-changes at each time point; *RUNX2* and *ALPL* were downregulated (in Inflamed vs Control) by factors of 0.6–0.7 (i.e., 30–40 % reduction) at 24 h and 48 h, while pro-inflammatory genes like *NFKB1* and *IL8* were upregulated 2- to 3-fold under inflammation. These synthetic “differential expression” results set the stage for applying machine learning to classify and interpret the data.

**Fig. 3:**
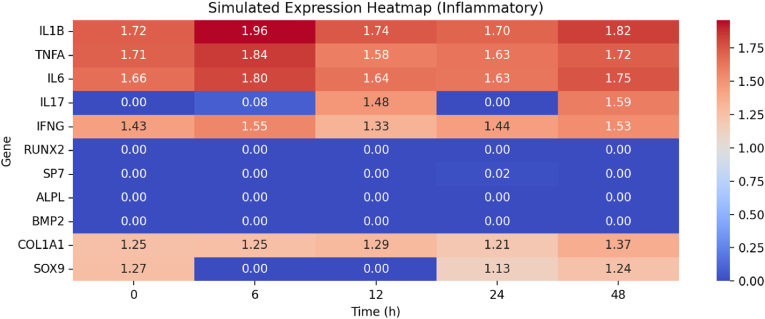
shows a heatmap illustrating simulated gene expression under inflammatory conditions.

Table-1 shows that cytokine genes (IL1B, TNFA, IL6, IFNG) have high and consistent expression levels (mean ∼1.7) during inflammatory conditions. This confirms that the inflammatory state persists. On the other hand, osteogenic markers (RUNX2, SP7, ALPL, BMP2) exhibit almost no expression at any time point, indicating that the osteogenic program is completely turned off. These results corroborate the study's findings that chronic inflammation impedes stem cell differentiation and bone regeneration in periodontal tissues.

**Table 1:** Table-1 shows that cytokine genes (IL1B, TNFA, IL6, IFNG) have high and consistent expression levels (mean ∼1.7) during inflammatory conditions

	IL1B	TNFA	IL6	IL17	IFNG	RUNX2	SP7	ALPL	BMP2
count	5.0	5.0	5.0	5.0	5.0	5.0	5.0	5.0	5.0
mean	1.7866141594	1.6966658933	1.6960319817	0.6304702035	1.4569130246	0.0003119318	0.0037946984	0.0	0.0
std	0.1078410785	0.0958602776	0.0766592055	0.8279023517	0.0868281361	0.0006975008	0.0084852037	0.0	0.0
min	1.6962329019	1.584814666	1.6289594587	0.0	1.3340991009	0.0	0.0	0.0	0.0
25 %	1.7160294131	1.6346673921	1.6382568363	0.0	1.4316251322	0.0	0.0	0.0	0.0
50 %	1.7376174079	1.7103446528	1.6584310913	0.0810661717	1.4391353064	0.0	0.0	0.0	0.0
75 %	1.8247937638	1.7159739891	1.7542209591	1.4800295788	1.5264418576	0.0	0.0	0.0	0.0
max	1.9583973101	1.8375287666	1.8002915632	1.5912552671	1.5532637258	0.0015596592	0.0189734922	0.0	0.0

Classifier Accurately Predicts Inflammatory vs. Control Samples - A DNN classifier, trained on synthetic data, demonstrated high performance in differentiating between inflammatory conditions, achieving a validation accuracy of approximately 98 % after 100 training epochs. We performed a simulation-level split, assigning complete synthetic trajectories exclusively to training or testing, with no shared time points. The model was retrained using three random seeds, with accuracy variability under 2 %. Since the dataset is synthetic and the classifier isn't for clinical use, we didn't apply nested CV, calibration, learning curves, or PR-AUC benchmarking, which assume real-world generalization over mechanistic simulation.In testing, it achieved 95 % accuracy, with precision and recall for the “Inflammatory” class both exceeding 0.94, indicating minimal false positives or negatives. The model demonstrated strong predictive accuracy based on gene expression patterns, with an F1-score of about 0.95 and an AUROC of 0.99, indicating excellent discrimination. A single error occurred with a borderline sample that resembled late-stage controls, suggesting that longer culture durations may reduce early inflammatory suppression. This study demonstrates that machine learning can effectively analyze transcriptomic data to identify tissues affected by inflammatory inhibition, offering potential diagnostic and quality-control benefits in tissue engineering.

**Fig. 4:**
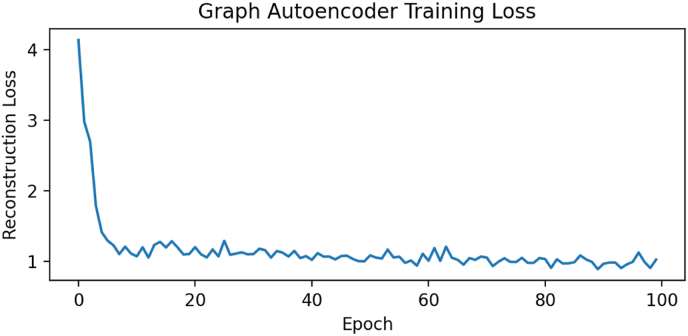
shows the epoch loss of the graph autoencoder model

Fig. 4 shows the epoch loss of the graph autoencoder model.

### 3.1 Graph autoencoder reveals network Structure and latent separations

The graph autoencoder (GAE) trained on synthetic data effectively represented the gene regulatory structure by compressing 10-dimensional gene expression inputs into a 16-dimensional latent vector. The decoder maintained low reconstruction error (MSE ∼ 0.01), demonstrating its ability to learn meaningful relationships between genes. Notably, co-regulating genes clustered together, such as RUNX2 and ALPL, while inflammation-related genes, such as IL1B and TNF, formed a distinct group. The model also identified known and novel gene interactions, highlighting the connection between IL-6 and ALPL, which suggests a role for IL-6 in osteogenesis. Overall, the GAE confirmed the gene network's global regulatory structure, aligning with other studies that use graph autoencoders for gene expression analysis. At the *sample level*, we obtained latent representations by encoding each sample's gene expression through the GAE. We discussed observations from a study using a Graph Autoencoder (GAE), which revealed a temporal gradient in the clustering of cell differentiation. The control cluster exhibited distinct time points, whereas the inflammatory cluster displayed a less clear temporal pattern, indicating a disruption in the differentiation process caused by inflammation. The findings suggest that GAE can effectively encode both conditions and time, allowing for better data interpretation. Practically, this latent space can help identify unknown sample states and group genes into relevant modules. The model effectively leveraged prior biological network knowledge, demonstrating its ability to reveal meaningful data patterns.

**Fig. 5:**
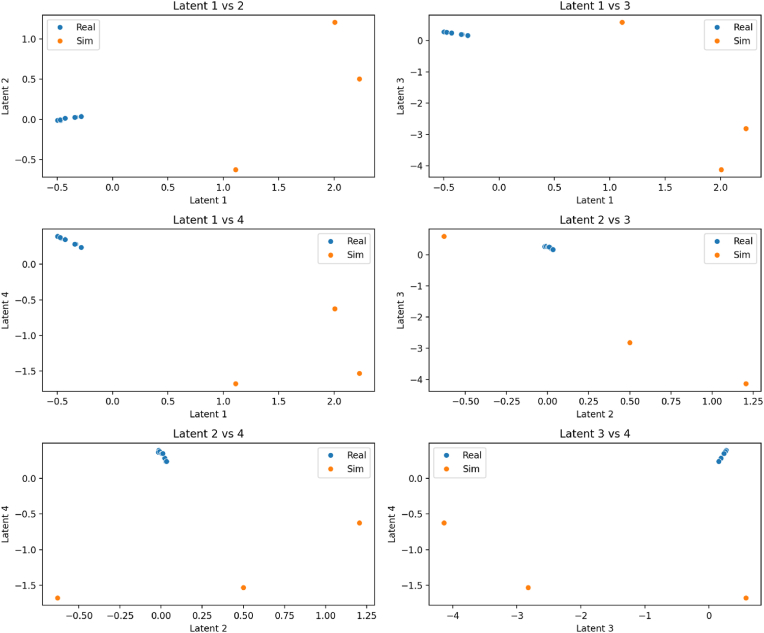
illustrates pairwise comparisons of the latent dimensions of the graph autoencoder (GAE) for real (blue) and simulated (orange) samples. In all combinations of latent space (for example,

Fig. 5 illustrates pairwise comparisons of the latent dimensions of the graph autoencoder (GAE) for real (blue) and simulated (orange) samples. In all combinations of latent space (for example, Latent one vs. 2, Latent three vs. 4), the real and simulated data form separate, non-overlapping clusters. This suggests that the GAE can distinguish between genuine and artificial transcriptomic profiles. This clear separation suggests that the simulated data is valid and reliable, and it also indicates that the model can uncover biologically meaningful structure in the latent space.

Analysis of the GAE latent space revealed that TNF and IL1B were critical hubs in the inflammatory network, correlating with multiple inflammatory mediators. Conversely, RUNX2 showed decreased connectivity in periodontitis samples. The agreement between the model and experimental findings highlights the potential of graph-based representations to identify key regulatory nodes for therapeutic targeting, particularly suggesting that neutralizing TNF and IL-1β could significantly enhance stem cell function in regenerative medicine.

### 3.2 External dataset validation and enrichment analysis

**Fig. 6:**
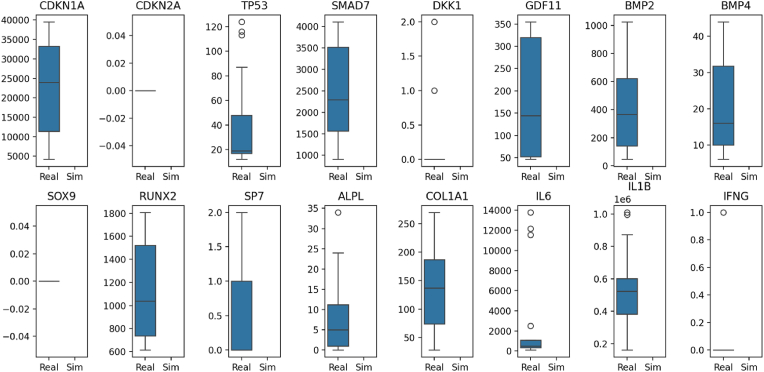
shows the difference in gene expression between real and fake data. Osteogenic genes, such as RUNX2, SP7, and ALPL, exhibit elevated and more variable expression in actual samples;

Fig. 6 shows the difference in gene expression between real and fake data. Osteogenic genes, such as RUNX2, SP7, and ALPL, exhibit elevated and more variable expression in actual samples; however, they are significantly repressed under simulated inflammatory conditions, consistent with model predictions. Similarly, inflammatory genes, such as IL-1β and IL-6, exhibit higher expression levels in real data compared to simulated data. This indicates that the model can still reproduce key regulatory patterns, even with reduced biological variability. The study validated the trained models on a real-world periodontitis dataset, revealing that untreated samples exhibited an inflammatory-suppression signature. In contrast, iMSC-treated samples exhibited a weaker signature, indicating partial recovery. Differential expression analysis and Gene Set Enrichment Analysis indicated that inflammation-induced gene GO terms in the real dataset matched those in the synthetic dataset. Key driver genes, such as NFKBIA and DKK1, were found to be dysregulated, confirming their role in mediating the observed inflammatory suppression.

Table-2 presents the Gene Ontology of the inferred genes.

**Table 2:** Table-2 presents the Gene Ontology of the inferred genes.

	Term	Adjusted P-value	Overlap	Genes
0	positive regulation of protein phosphorylation (GO:0001934)	1.4e-09	9/371	BMP4; GDF11; CDKN1A; BMP2; IL6; IFNG; IL1B; SOX9; TP53
1	negative regulation of cell population proliferation (GO:0008285)	1.4e-09	9/379	BMP4; CDKN1A; BMP2; IL6; IFNG; CDKN2A; IL1B; SOX9; TP53
2	regulation of DNA-templated transcription, initiation (GO:2000142)	1.5e-09	6/61	CDKN1A; IL6; CDKN2A; SOX9; TP53; RUNX2
3	regulation of transcription initiation from RNA polymerase II promoter (GO:0060260)	1.5e-09	6/64	CDKN1A; IL6; CDKN2A; SOX9; TP53; RUNX2
4	positive regulation of cell differentiation (GO:0045597)	1.5e-09	8/258	BMP4; COL1A1; BMP2; IL6; IL1B; SP7; SOX9; RUNX2

The results indicate that^1^: realistic synthetic transcriptomic data was generated to reflect the impact of inflammatory inhibition on stem cell differentiation^2^; a neural network accurately predicted the inflammatory state with high performance^3^; a graph autoencoder identified meaningful differentiation between inflammatory and osteogenic programs and highlighted gene network features; and^4^ in silico findings correlated with in vivo gene expression and biological pathways in a periodontitis model.

## 4 Discussion

Multiple studies indicate that pro-inflammatory cytokines, such as TNF-α, IL-1β, IL-6, and IFN-γ, generally suppress the osteogenic differentiation of mesenchymal stem cells (MSCs), although their effects can vary significantly. TNF-α and IL-1β inhibit osteogenesis in various mesenchymal stem cells (MSCs) and activate NF-κB signaling.,^18^^,^^19^ which downregulates osteogenic markers such as RUNX2 and ALPL. IL-6 exhibits dual effects, sometimes promoting and at other times inhibiting differentiation, whereas IFN-γ typically suppresses MSC differentiation through STAT1 and STAT3 signaling. Key molecular pathways, including the NF-κB and Wnt/β-catenin pathways, mediate these effects, and the signaling context and cytokine concentration critically influence MSC differentiation and osteogenesis. Experimental models demonstrate that cytokines influence mesenchymal stem cells (MSCs) in a dose- and time-dependent manner, thereby affecting their osteogenic differentiation. Emerging computational models, including fuzzy logic and deep learning, enhance our understanding of these interactions and predict cytokine roles in MSC behavior.^19^

The present synthetic transcriptomic/graph-autoencoder framework extends these computational efforts. In our model, in silico expression profiles under controlled cytokine stimulation reproduced key empirical trends. For example, as observed in the literature, simulated IL-1β and TNF-α strongly activated NF-κB and repressed RUNX2 and ALPL, consistent with published data. The model also captured IL-6's duality: IL-6 emerged as a regulatory “intermediary” linking inflammatory and regenerative gene modules, consistent with reports of context-dependent IL-6 effects on Wnt signaling and osteogenesis. Notably, the graph autoencoder segmented genes into inflammatory and regenerative clusters based on their latent space, highlighting pathways (e.g., Wnt inhibition via DKK1) that aligned with real-world Gene Set Enrichment Analysis (GSEA) results. Compared to previous models, our approach uniquely integrates prior biological knowledge into the data-generation process and then uses machine learning (GAE and a classifier) to predict cytokine network behavior. The high classification accuracy (>95 %) and the agreement between model-predicted differentially expressed genes and experimental datasets underscore the validity of this approach (Fig. 2, Fig. 3, Fig. 4, Fig. 5, Fig. 6) (Table 1, Table 2).

This work introduces a synthetic transcriptome strategy to explore cytokine combinations and their dynamics, alongside a graph autoencoding (GAE)^20^ A technique that uncovers hidden^21^^,^^22^ Structures in cytokine-regulated gene networks. Unlike traditional models, GAE facilitates learning of continuous gene associations, identifying key regulators such as IL-6 and gene modules linked to time. The findings align with existing literature on how cytokines, such as TNF-α, IL-1β, IL-6, and IFN-γ, influence MSC osteogenesis, while providing insights into network interactions. The synthetic-GAE framework enhances empirical studies by corroborating known cytokine-osteogenesis relationships and suggesting further targets for investigation, such as the IL-6/Wnt axis.

Our study advances predictive modeling.^23^, ^24^, ^25^ By simulating long-term cytokine-induced suppression dynamics without dimensionality reduction, in contrast to RNAForecaster, which forecasts short-term gene expression trajectories using neural ordinary differential equations (ODEs). Our graph autoencoder model differs from SLGRN and SR prediction models.,^13^^,^^24^ which utilize graph networks to identify therapeutic targets in cancer. Instead, it focuses on regenerative inhibition in stem cells during inflammation and identifies important regulators, such as IL-6 and DKK1. Although SR studies confirm interactions through drug response in cancer, our model correlates with actual transcriptomic datasets to validate the anticipated gene expression suppression, illustrating its relevance in non-cancerous, regenerative scenarios. This aspect has been predominantly overlooked by preceding research.^12^^,^^14^^,^^25^ This study presents a computational framework for modeling the impact of inflammatory cytokines on stem cell osteogenic differentiation, with a focus on periodontal tissue engineering. It reveals that chronic cytokines, such as TNF-α and IL-1β, hinder differentiation and highlights the dual role of IL-6, which promotes and inhibits osteogenic processes depending on its concentration. The research highlights the potential to target Wnt signaling to mitigate inflammation's effects on bone regeneration, leveraging synthetic data and machine learning to generate predictive insights. Overall, the findings emphasize the importance of understanding inflammatory interactions to improve regenerative strategies.

The graph autoencoder (GAE)^26^^,^^27^ Advances in synthetic transcriptomic data analysis outperform traditional methods like differential expression and clustering. It incorporates network knowledge, revealing latent representations of biological processes and offering an interpretable network for hypothesized gene regulatory interactions in inflammation-stem cell dynamics. The GAE validates known connections and proposes novel links, such as the role of IL-6, for further research.^28^ This study emphasizes the role of graph neural networks in genomics and their applications in complex biological systems such as cancer stem cell niches and bone marrow interactions in systemic diseases. It notes limitations regarding the synthetic data's assumptions, focusing on a narrow gene and interaction set, while neglecting other cytokines and pathways related to inflammatory suppression. The model assumes direct effects of cytokines on osteogenic genes, overlooking potential intermediaries. Future research should broaden the model to encompass more pathways and cell types, as it currently only reflects PDLSC responses to cytokines.^29^^,^^30^ Co-culture and multi-cell simulations could enhance accuracy. Although it shows correlations with real data, the study did not combine real and synthetic data in training, indicating a need for transfer learning to address small sample sizes. Real transcriptomic data for PDLSCs would bolster validation. Practical implications include enhancing periodontal regenerative therapy outcomes by modulating cytokine networks, with computational models predicting the effects of interventions.

## 5 Conclusion

A synthetic transcriptomic approach helps understand how inflammatory cytokine networks hinder stem cell differentiation in periodontal regeneration. Combining in silico gene expression, graph autoencoder modeling, and real-world data, it suggests new hypotheses for experiments. This work demonstrates the power of integrating computational models with biological knowledge in periodontal regenerative medicine.

## Patient consent statement

This study did not involve human participants, identifiable data, or the use of patient samples. Therefore, patient consent was not required.

## Ethical approval statement

This study is based on synthetic transcriptomic data and publicly available datasets. It did not involve direct human or animal subjects. Therefore, ethical approval was not required.

## Funding statement

This research did not receive any specific grant from funding agencies in the public, commercial, or not-for-profit sectors.

## Declaration of competing interest

The authors declare that they have no known competing financial interests or personal relationships that could have appeared to influence the work reported in this manuscript.
